# Measuring adherence to ARVs among HIV-positive adolescents in Cameroon: a comparative assessment of self-report and medication possession ratio methods

**DOI:** 10.11604/pamj.2021.40.148.27994

**Published:** 2021-11-10

**Authors:** Mbuwir Charlotte Bongfen, Kwasi Torpey, John Ganle, Ankomah Augustine

**Affiliations:** 1 Biaka University Institute Buea, Buea, Cameroon,; 2 Department of Population, Family and Reproductive Health, School of Public Health, University of Ghana, Legon, Accra, Ghana

**Keywords:** Adherence measurements, adolescents, self-report, medication possession ratio, viral load suppression

## Abstract

**Introduction:**

adherence to ARV medications has been shown to improve treatment outcomes in HIV positive patients. Given that ARV treatment is lifelong, adherence has become a critical issue as it may reduce over time. Measuring adherence is therefore imperative in programming. There are different methods of measuring adherence each with its advantages and disadvantages, depending on the context and the time. This study therefore compares two widely used adherence measurement scales in Cameroon, namely, the self-report and the medication possession ration (MPR) methods.

**Methods:**

the study was done in some selected health facilities of the North West and South West regions of Cameroon among adolescents on ARV. The study was designed as an analytical cross-sectional study with a record review component and systematic random sampling was used to select the participants. Adherence was measured through self-report and the medication possession ratio. Adolescents with adherence levels of at least 95% were considered adherent. Viral load suppression was considered as having the most recent viral load suppression results of less than 1000 copies per ml. The kappa statistics of inter-rate agreement was used to ascertain the difference between adherence as measured by self-report and MPR. The difference in adherence between the two scales was also compared using Fischer´s exact test and p-values were reported.

**Results:**

the study shows that adherence level using the self-report technique is 82.9% while that of MPR was 73.4%. When compared using the using Kappa statistics, there was substantial agreement between the two scales of 66% (p=0.54). The results of both self-report adherence and MPR were also compared with viral load suppression and the difference between viral load suppression and MPR was significant (p<0.01). The difference in adherence between viral load suppression and the self-report measure also showed to be significant (p<0.01).

**Conclusion:**

adherence from the self-report measure was higher than from MPR, but there was substantial agreement between the scales. Although there is no gold standard for adherence measurement, self-report or medication possession ratio could be used and complemented with laboratory markers like viral load counts.

## Introduction

Adolescents and young people contribute a huge proportion of people living with HIV globally. Generally, young people and adolescents have been more susceptible to HIV infection. By June 2018, it was estimated that, out of the 37.9 million people living with HIV (PLHIV) worldwide, more than 90% were in the global south, with 1.7 million of them being aged less than 15 years [1]. United Nations children's fund (UNICEF) estimated 170,000 new HIV infections among adolescents in 2019 alone [2]. The prevalence of HIV among adolescence in Cameroon was estimated to be 2% in 2015, with about new 4200 infections and 1900 deaths [3]. As of 2016, a total of about 40,000 adolescents were living with HIV in Cameroon [4]. Globally, adherence has been estimated to be lower in adolescents than in other age groups [5]. A systematic review by Sung-Hee and others in 2014, reported adherence in adolescents to be 62.3% [5]. In Cameroon self- report adherence to ARVs among adolescents was estimated at 36% in a study in Yaounde by Fokam and others (Fokam *et al*.2017).

All HIV patients are on daily medication and the process is difficult and monotonous such that after six months, adherence to the medications begin to drop as shown in a study by Nsheha and others in 2014 [6]. Given the importance of adherence therefore, it is necessary to take a closer look at adherence and its measurements. This study has hence measured and compared adherence among adolescents using self-report and medication possession ratio (MPR).There are varied methods of measuring adherence with advantages and disadvantages depending on the context and the time [7,8]. In the absence of directly observed therapy (DOTS), the levels of adherence can only be estimated using other available measures [9]. Some of the available indirect methods of measuring adherence include; self-reports, electronic drug monitoring (EDM), pill counts and pharmacy refill records to obtain medication possession ratio [10]. Adherence can also be measured directly by measuring metabolites including detection of drugs in plasma. However, there is no gold standard of adherence measurement [11] although self-report is the most used tool for adherence measurement [12].

**Objective:** the objective of this study is therefore to compare two widely used adherence measurement scales in Cameroon, namely, the self-report and the medication possession ration (MPR) methods.

## Methods

**Study setting:** the study was done in some selected health facilities of the North West and South West regions of Cameroon. This study targeted adolescents (10-19 years) who were living with HIV and aware of their status and had been on treatment for at least 6 months. Data for this study was collected between September 2018 and February 2019.

**Study design and sampling:** the study was designed as an analytical cross-sectional study with a record review component. The records of the sampled adolescents were reviewed to obtain viral load results. A total of 9 health facilities were purposively selected based on the case load of the number of adolescents registered on treatment. This data was based on information obtained from the HIV regional technical group (RTG) for the North and South West Region. Probability proportionate to size sample allocation was used to obtain the number of adolescents per site. Systematic random sampling was then used select the participants in each of the sampled health facilities and a total of 460 respondents were recruited from the nine health facilities.

**Sample size:** the sample size of the study is 460. This was estimated using the Cochrane´s formula for calculating sample size for cross-sectional studies.


$$n=\frac{Z^{2}P\left(1-p\right)}{d^{2}}$$

Where; n= minimum sample size required for the study; Z^2^= critical value, 1.96 P= expected level of adherence (36%); d=precision, which was set at 0.05. The expected level of adherence (self-report) (36%) used was obtained from a 2017 study in Cameroon that measured adherence among adolescents (Fokam et al., 2017). Based on the assumptions above, the minimum sample size estimated was 354. A non-response rate of 30% was assumed so as to increase the power of the study, and this gave a total sample size of 460.

**Variables and measurements:** for the purpose of this study, adherence was defined as the patient´s ability to take medications as per the prescription. Firstly, it was determined through self-report of last missed pills. Self-report adherence was measured based on the 30 days recall. It was calculated as the proportion of pills taken to the number prescribed within 30 days. For example, a patient on one pill daily could have missed 2 pills in the past 30 days, hence the adherence of that participant will be 28/30 = 93%. Based on this premise, participants with a self-report score of≥ 95% were considered adherent. The data on the number of pills missed in the last 30 days to calculate adherence was obtained from a structured questionnaire which was interviewer administered. Adherence was also measured through pharmacy records. This calculation from pharmacy records was done based on the medication possession ratio (MPR). Medication possession ratio was calculated as the sum of the days of treatment supplied for all ART prescriptions filled, within the refill interval divided by the number of days during that same time period. A patient was considered as adherent if the MPR was at least 95%. Adherence was coded as a binary outcome variable. Only results of respondents who had results for both MPR and self- report were used to compare adherence between the two measures. Viral load suppression was considered as having the most recent viral load suppression results of less than 1000 copies per ml. The most recent viral load had to be within the past 6 months. Participants with less than 1000 copies/ml were considered having suppressed their viral loads. This viral load was extracted from patient´s records in the health facility. All the viral load results done were obtained with the dates on which they were done.

**Statistical analysis:** the kappa statistics of inter-rater agreement on a nominal scale was used to ascertain the agreement between adherence as measured by self-report and MPR. The scale is interpreted as shown on Table 1 [13]. Based on the kappa statistics, individual adherence measures of the participants for both self-report and MPR were subjected to a kappa test on STATA 15 and the agreement level obtained and interpreted as per the scale on Table 1. Furthermore, the viral load results were also used as a standard to compare adherence as measured by self-report and MPR. The difference in adherence between the two scales was compared using Fischer´s exact test and p-values were reported. All these analyses were aided by STATA 15.

**Table 1:** the Kappa benchmark scale

Agreement level	Interpretation
0.00	Poor agreement
0.0-0.20	Slight agreement
0.21-0.40	Fair agreement
0.41-0.60	Moderate agreement
0.61-0.80	Substantial agreement
0.81-1.00	Almost perfect agreement

**Bias:** the self-report method for measuring adherence was used in this study. Therefore, there was the possibility of participants overestimating adherence. However, there was significant agreement between the self-report measure and the medication possession ratio, which suggests that the issue of overestimation may have been minimised.

**Ethics approval and consent to participate:** ethical approval was received from the Cameroon Baptist Convention (CBC) ethical review board (IRB2018-41). Administrative approval was obtained from the Regional Delegation of Health at the North and South West Regions of Cameroon. During data collection, written informed consent was obtained from adolescents who were 18 years and above and from the guardians of adolescents who were less than 18 years. Written assent was then obtained from adolescents less than 18 years.

## Results

**Participants:** out of the 460 questionnaires administered, 455 were returned. For the self-report adherence, 405 adolescents responded while for the MPR data was collected for 418 adolescents. As for the viral load counts, data was available for 419 adolescents.

**Descriptive data:** there were more females (55%) than males (45%). The mean age of the participants was 14.8years (SD = +2.9). A greater proportion of the adolescents (43%) were young (10-14years).Results on clinical characteristics of participants indicated that 82% were on first line regimens of ARVs, while 18% were on second line. Only 3 of the participants (0.7%) were on third line regimen. The average duration of treatment was 67.3months (SD= ±46.6)

### Main results

**Self- report ARV adherence:** as noted earlier, self-report ARV adherence was measured based on a 30-day recall of pills taken. Overall, 336 (82.7%) of the participants were adherent to ARV treatment and the remaining 69 (17.0%) were non-adherent. Hence, the self-report measure of adherence was 83.0%.

**Medication possession ratio (MPR):** a total of 418 adolescents had data for MPR. From the results obtained with the MPR formula, 307 (73.4%) of the participants were adherent whereas 111 (26.6%) of them were non-adherent.

**Relationship between self-report and MPR:** adherence was shown through MPR to be lower than the self- report measure (Table 2). The relationship between the self-report measure and MPR was assessed. Only the participants who had data for both MPR and self-report were considered. A total of 82 participants had missing data on both MPR and self-report adherence. The Kappa statistics was used to assess the level of agreement between the two measures. The level of agreement was 66% (p = 0.54).Based on the Kappa scale, the 66% agreement observed indicated that there is substantial agreement between self-report and the medication possession ratio adherence.

**Table 2:** relationship between self-report adherence measure and medication possession ratio in a group HIV-positive adolescents in nine health facilities of the North West and South West Regions of Cameroon between September 2019 and February 2020(N=460)

Medication possession ratio			
**Self-report**	**Adherent n (%)**	**Non-adherent n (%)**	**Total**
Adherent n (%)	232(74.8)	78(25.2)	310
Non-adherent n (%)	47(74.6)	16(25.4)	63
Total	279	94	373

**Relationship between adherence and viral load:** having a suppressed viral load may be a strong indication of good adherence. From the analysis, 215 (61.0%) of the adolescents who were adherent had a suppressed viral load. The relationship between self-report and MPR with viral load suppression were assessed. The difference in viral load suppression between those who were adherent for both self-report and MPR and those who were not adherent was significant (p=0.03). Table 3 shows the relationship between viral load suppression and adherence.

**Table 3:** relationship between adherence and viral load suppression among adolescents in the North West and South West region of Cameroon between September 2019 and February 2020 (N=460)

Type of Adherence measure	Viral load suppressed n (%)	Viral load not suppressed n (%)	P-value
**Self-report**			
Adherent(Ref)	215(73.6)	77(26.4)	-
Non-adherent	30(52.6)	27(47.4)	<0.01
**medication possession ration (MPR)**			
Adherent(Ref)	189(79.2)	75 (20.8)	-
Non-adherent	25 (39.9)	41 (60.1)	<0.01

The p-values depicts the level of significance between each measure of adherence and viral load suppression

## Discussion

**Key results:** adherence was also measured through the medication possession ratio (MPR) and the results were lower than the self-report measure. The self-report adherence was 83% while adherence based on medication possession ratio (MPR) was 73.4%.

**Interpretation:** the difference between the two measures (self-report and MPR) was compared using the Kappa statistics. There was substantial agreement of 66% between the two measures. This suggests that the self-report and MPR method of measuring adherence is likely to yield similar results if used in the same population under the same or similar context. Indeed, this study is one of the few studies to compare adherence measurements using self-report and MPR among adolescents and has shown some concordance between the two scales. Earlier studies have compared pill counts and viral load suppression, which showed little agreement(16%) between the two scales [14]. A study by Denison and others in 2015 had reported adherence using self-report and MPR but did not show the level of agreement between the two methods [15].

Further analysis showed that, only 73.6% of the adolescents who were adherent (self-report) had suppressed viral load. In the case of MPR, 79.2% of the adherent adolescents had suppressed viral load. This confirms the trend that adherence to ARV treatment is a main predictor of viral suppression. This result were slightly higher than those of Chikwari in Zimbabwe that reported viral load suppression of 63% among adherent adolescents [16]. These findings also suggest that MPR may be more predictive of viral load suppression compared to self-report. An earlier study had also shown that pharmacy refill records could suggest HIV virologic failure [17]. The general limitation of the study is the fact that there were missing data as since were some respondents who did not have data for self-report, MPR and viral load suppression and hence could not be included in the final analysis comparing adherence and viral load suppression. There were also a proportion of adolescents who were non-adherent but had suppressed viral loads. This is possible given that the newer regimens are more forgiving an adherence of 95% may not be absolutely needed to suppress the virus.

**Limitations:** the study reported a proportion of adolescents who were non-adherent but had suppressed viral loads. It has been suggested that this kind of discrepancy is also possible because even in the case of resistance, ARV drugs regimens still exert some degree of anti-HIV activity on the resistant virus [18]. Furthermore, adherence levels between 50% to 100% can achieve viral load suppression when using non-nucleoside reverse-transcriptase-inhibitors (NNRTIs) regimens [19]. Findings from the study also revealed that 22% of adolescents who were adherent to treatment had unsuppressed viral loads. This is counterintuitive as the expectation is that, being adherent, should lead to viral load suppression. This could probably be attributed to undiagnosed treatment failure and resistance, or the fact that some of the adolescents might have over reported self-report adherence [20]. This is an indication that adherence reports in health settings should be complimented by laboratory markers like viral load to reduce possibilities of unnoticed resistance to treatment, which could gradually aggravate to AIDS.

## Conclusion

Adherence from the self-report measure was higher than from MPR, but there was substantial agreement between the scales. It is therefore recommended that for practice, self-report or medication possession ratio could be used for adherence measurement as there is substantial agreement between them. However, this should be complemented with laboratory markers like viral load counts. A further study can also be done to ascertain the situation in adults as the study was limited to adolescents whose adherence pathways might be different from those in adults.

### What is known about this topic

- Earlier studies have compared pill counts and viral load suppression;
- A study that had reported adherence using self-report and MPR did not show the level of agreement between the two methods.

### What this study adds

- The study has shown the level of agreement (Substantial agreement) between self-report measurements and medication possession ratio;
- The study also compared the adherence measurements with viral load counts and there were discrepancies, therefore indicating need to complement adherence measurements (self-report and MPR) with laboratory markers like viral load counts in clinical practice;
- This is also the first study in the Cameroonian context comparing these two methods of adherence measurements among adolescents.
